# Micelle-stabilized Olfactory Receptors for a Bioelectronic Nose Detecting Butter Flavors in Real Fermented Alcoholic Beverages

**DOI:** 10.1038/s41598-020-65900-6

**Published:** 2020-06-03

**Authors:** Narae Shin, Seung Hwan Lee, Viet Anh Pham Ba, Tai Hyun Park, Seunghun Hong

**Affiliations:** 1 Department of Physics and Astronomy, and Institute of Applied Physics, Seoul National University, Seoul, 08826 Korea; 2 School of Chemical and Biological Engineering, Institute of Chemical Processes, Seoul National University, Seoul, 08826 Korea; 3 Department of Bionano Engineering and Bionanotechnology, Hanyang University, Ansan, 15588 Korea; 4 Department of Environmental Toxicology and Monitoring, Hanoi University of Natural Resources and Environment, Hanoi, Vietnam

**Keywords:** Nanoscale biophysics, Biosensors

## Abstract

A bioelectronic nose device based on micelle-stabilized olfactory receptors is developed for the selective discrimination of a butter flavor substance in commercial fermented alcoholic beverages. In this work, we have successfully overexpressed ODR-10, a type of olfactory receptor, from *Caenorhabditis elegans* using a bacterial expression system at a low cost and high productivity. The highly-purified ODR-10 was stabilized in micelle structures, and it was immobilized on a carbon nanotube field-effect transistor to build a bioelectronic nose for the detection of diacetyl, a butter flavor substance, *via* the specific interaction between diacetyl and ODR-10. The bioelectronic nose device can sensitively detect diacetyl down to 10 fM, and selectively discriminate it from other substances. In addition, this sensor could directly evaluate diacetyl levels in a variety of real fermented alcoholic beverages such as beer, wine, and makgeolli (fermented Korean wine), while the sensor did not respond to soju (Korean style liquor without diacetyl). In this respect, our sensor should be a powerful tool for versatile food industrial applications such as the quality control of alcoholic beverages and foods.

## Introduction

Diacetyl (2,3-butanedione) is a *buttery flavor* compound that exists in various foods and beverages such as beer, wine, butter, milk, and yogurt^1,2^. This compound is the byproduct of fermentation processes by some species of microorganisms such as bacteria and yeast. One of the foremost criteria for the evaluation of fermented foods is butter flavors, which influences consumer acceptance. Consumers of fermented foods appreciate such a buttery flavor. These characteristic traits and the quality are affected by diacetyl concentrations. However, diacetyl may impart a negative effect on food flavors when its concentration is higher than the odor threshold. Also, the hyperingestion of diacetyl can cause undesirable diseases such as a lung disease, Alzheimer, bronchiolitis, and obliterans syndrome^1–5^. Thus, it is quite important to measure the concentration of diacetyl for the quality control of various food products. In recent decades, various analytical methods to detect diacetyl have been developed including spectrophotometry, a gas chromatograph-mass spectrometer (GC-MS), and high-performance liquid chromatography (HPLC)^6–15^. However, these conventional methods have some limitations. For instance, the spectrophotometry method is simple, but usually exhibits rather low sensitivity and selectivity^11^. The analysis methods based on GC-MS and HPLC can be used to detect diacetyl with a high sensitivity, while requiring rather long complicated processing steps with a high cost^12,14^.

On the other hand, several researchers have attempted to develop a sensor system utilizing biomaterials as probes for the detection of food flavor substances^16,17^. For example, ODR-10 odorant receptors are a member of G protein-coupled receptors (GPCR) involved in diacetyl chemotaxis in *Caenorhabditis elegans* (*C. elegans*)^9^. ODR-10 receptors were expressed on cells and used for the detection of diacetyl through the combination with a surface plasmon resonance spectroscopy^10^. However, such cell-based sensor system often exhibited a rather low sensitivity compared with conventional analysis methods. Extensive efforts have been given to extract only GPCR proteins from *Escherichia coli* (*E. coli*) and to combine them with sensor transducers to build a compact sensor device. For example, quartz crystal microbalance (QCM) devices have been combined with extracted ODR-10 proteins to build a bioelectronic nose device for the detection of food flavor substances^18^. However, such ODR-10 protein-based probes for sensor systems may be unstable in some real-food environments such as alcoholic beverages. For example, the instability of the GPCR protein under complex real-food environments could lead to non-specific binding of the protein and, eventually, the low selectivity of biosensor devices based on it like other protein-based biosensors^19,20^. To enhance its stability, extensive studies were carried out including the purification and functional reconstitution of GPCRs in general^21,22^. Among them, a detergent micelle structure has been widely used for the reconstitution of stable GPCRs. In this strategy, GPCRs were packed tightly in amphiphilic-phospholipids so that they can form a stable structure in aqueous environments and mimic the native structure of the GPCRs in a cell. Such micelle-stabilized GPCRs have been utilized to build stable biosensor devices based on various sensor transducers^21,22^. However, such ODR-10 stabilized in micelle structures has not been applied to build bioelectronic nose devices.

Herein, we report micelle-stabilized ODR-10 olfactory receptors for a bioelectronics nose discriminating a butter flavor substance in real-fermented alcoholic beverages. In this work, an olfactory receptor protein of *C. elegans*, ODR-10, was overexpressed in *E. coli* and functionally reconstituted in detergent micelles for stability in an aqueous environment. Then, the reconstituted ODR-10 in detergent micelles was successfully immobilized on the channel region of a carbon nanotube (CNT) field effect transistor, enabling a bioelectronic nose, an artificial olfactory sensor system. Our CNT-based bioelectronic nose device could detect a butter flavor substance, diacetyl, down to 10 fM, indicating that our sensors are more sensitive than a human sensory system^23^. Presumably, it is because, in a human sensory system, the binding event of odorant molecules onto olfactory receptors should trigger multiple chemical processes to be transmitted to a human brain, while, in our sensor, the activity of receptor protein was directly measured by a very sensitive CNT-based transducer. Similar results were obtained in our previous works about bioelectronic nose devices based on other receptor proteins^16,17,24,25^. Furthermore, we could utilize our devices to quantitatively evaluate the diacetyl substance directly in real-alcoholic beverages such as beer, wine, and makgeolli (fermented Korean wine). Since our method allows one to quantitatively evaluate the butter flavor substance in real-samples with a rather simple manner compared with previous methods such as fluorescence assays and HPLC^9,12^, it can be a powerful tool for various basic research and industrial applications such as the screening of alcoholic beverages and foods.

## Results and Discussion

### Schematic diagram showing the structure of a CNT-based bioelectronic nose

**Figure 1:**
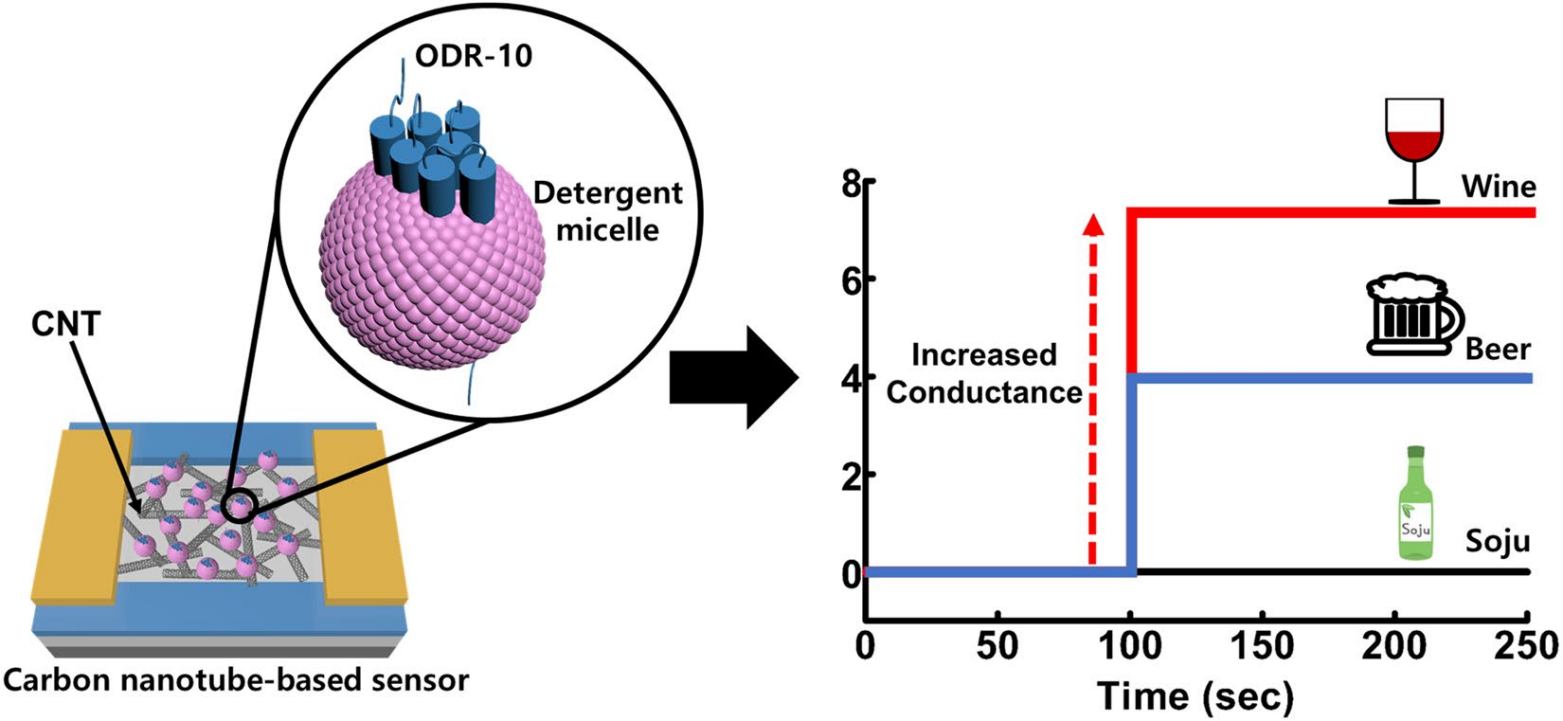
Schematic diagram showing the structure of a CNT-based bioelectronic nose, and the simplified responses of the sensor to diacetyl in commercial alcoholic beverages. The CNT-FET was fabricated by previously-reported processes including photolithography and thermal evaporation methods. The micelle-stabilized ODR-10 receptors were immobilized on the CNT channel using PSE molecules as a linker.

Figure 1 shows a schematic diagram depicting a CNT-based bioelectronic nose with micelle-stabilized ODR-10 and its electrical responses. The detailed fabrication process is provided in the Methods section. In brief, the diacetyl receptor of *C. elegans*, ODR-10, was overexpressed in a bacterial expression system such as *E. coli*^18^. Then, ODR-10 was functionally reconstituted and stabilized in detergent micelles^26^. The CNT channel region was coated with 1-pyrenebutanoic acid succinimidyl ester (PSE) as a non-covalent linker^17^. Lastly, the ODR-10 receptors were selectively immobilized onto the CNT channel region. For the sensing experiment, sample solutions including diacetyl with different concentrations were applied to a bioelectronic nose device while monitoring the conductance change of the CNT channels in the device. When diacetyl bound to the ODR-10 receptors, the charge state of ODR-10 was altered and the conductance of the CNT channel was changed, allowing us to monitor diacetyl in real-time.

### Characterization of ODR-10 receptors expressed in *E. coli* and HEK-293 cells

**Figure 2:**
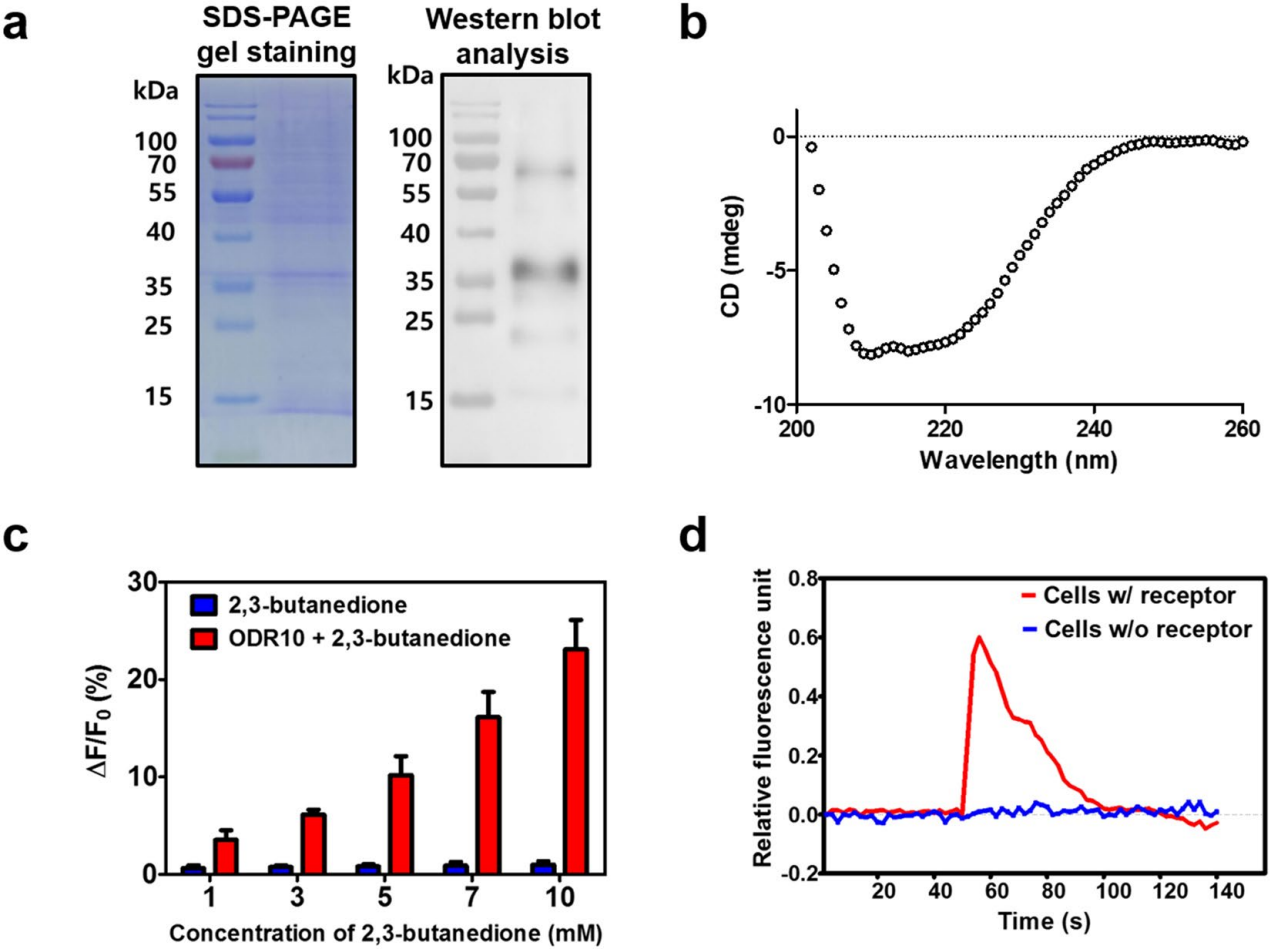
Characterization of ODR-10 receptors expressed in E. coli and HEK-293 cells. (**a**) SDS-PAGE and western blot analysis of purified ODR-10 produced in E. coli. The V5 antibody was used for western blot analysis. (**b**) Circular dichroism spectrum of reconstituted ODR-10 in detergent micelles. (**c**) Tryptophan fluorescence quenching assay of ODR-10 with a dose-dependent stimulation of diacetyl (**d**) Real-time fluorescence measurement of HEK-293 cells expressing ODR-10 in response to diacetyl stimulation. The images of gel and blot are used in (a) were cropped from different image. To clarify this, the cropping lines were expressed by the black lines surrounding gel and blot and full-length gel and blot are included in Supporting Information (Fig. S1 in Supporting Information).

To construct structurally and functionally high-quality receptor proteins, ODR-10 was overexpressed in *E. coli*, purified, and reconstituted in detergent micelles^26^. Detailed procedures for expression, purification and reconstitution of ODR-10 are described in the Methods section, and full-length gel and blot are included in Supporting Information (Fig. S1 in Supporting Information). These processes are confirmed using the sodium dodecyl sulfate-polyacrylamide gel electrophoresis (SDS-PAGE) (left) and the western blot analysis (right) (Fig. 2a) (see Methods section for details). The SDS-PAGE gel showed that ODR-10 was successfully purified to high purity. Expression of ODR-10 was confirmed using western blot analysis with the V5 antibody. The bands of SDS-PAGE and western blot were well matched with the molecular weight of ODR-10^9^. These results indicate that ODR-10 was well expressed and purified to high purity.

The reconstitution of ODR-10 in detergent micelles was evaluated using circular dichroism (CD) spectra (Fig. 2b). Detailed procedures for CD spectrum measurements are described in the Methods section. In the CD spectroscopy, the amide chromophores of the peptide bonds in the proteins cause a change in the optical transitions of circular polarized light. This change results in characteristic CD spectra of proteins between wavelengths of 190 and 240 nm^26^. Based on this, the CD spectrum analysis of reconstituted ODR-10 in detergent micelles was conducted at 190 to 260 nm. As shown in Fig. 2b, “w”-shaped spectra with two negative peaks at approximately 210–220 nm and a larger positive peak at approximately 190 nm were observed. This result indicates that our reconstitution process led to the successful construction of the secondary structure of ODR-10^26,27^.

The functional affinity of ODR-10 for diacetyl was analyzed using tryptophan fluorescence quenching assay (Fig. 2c). Tryptophan residues in proteins show intrinsic fluorescence observable at emission wavelengths of 340 to 350 nm^28^. The fluorescence signals are quenched because the structures of the proteins are transformed as the target molecules bind to the receptor proteins. In this study, the stimulation of reconstituted ODR-10 in detergent micelles at various diacetyl concentrations induced a conformational change of ODR-10, which shielded the tryptophan residues and quenched their fluorescence signal. The change in the fluorescence signals for reconstituted ODR-10 in detergent micelles increased as the concentration of diacetyl increased, in a dose-dependent manner. However, the change in fluorescence did not occur in the absence of ODR-10. These results indicate that ODR-10 responded selectively to diacetyl in a dose-dependent manner because the receptors were well reconstituted and stabilized in detergent micelles through the reconstitution process.

Figure 2d shows the real-time profiles of Ca^2+^ in HEK-293 cells expressing ODR-10. A calcium indicator dye, Fluo-4 AM, was loaded into the HEK-293 cells expressing ODR-10, following which, the cells were stimulated with diacetyl (1 mM) (see the details in the Methods section). The fluorescence signal of the cells immediately increased as a result of cell stimulation, due to the binding of diacetyl to ODR-10 following a Ca^2+^ release into the cytosol. The fluorescence signal of the calcium indicator recovered to the baseline level as Ca^2+^ concentration was restored by ion pumps^16^. However, cells transfected with the mock vector did not show the change in the fluorescence signal with diacetyl stimulation. These results clearly show that ODR-10 was expressed on the surface of cells and recognized the diacetyl.

### Responses of CNT-based bioelectronic noses to diacetyl solutions

**Figure 3:**
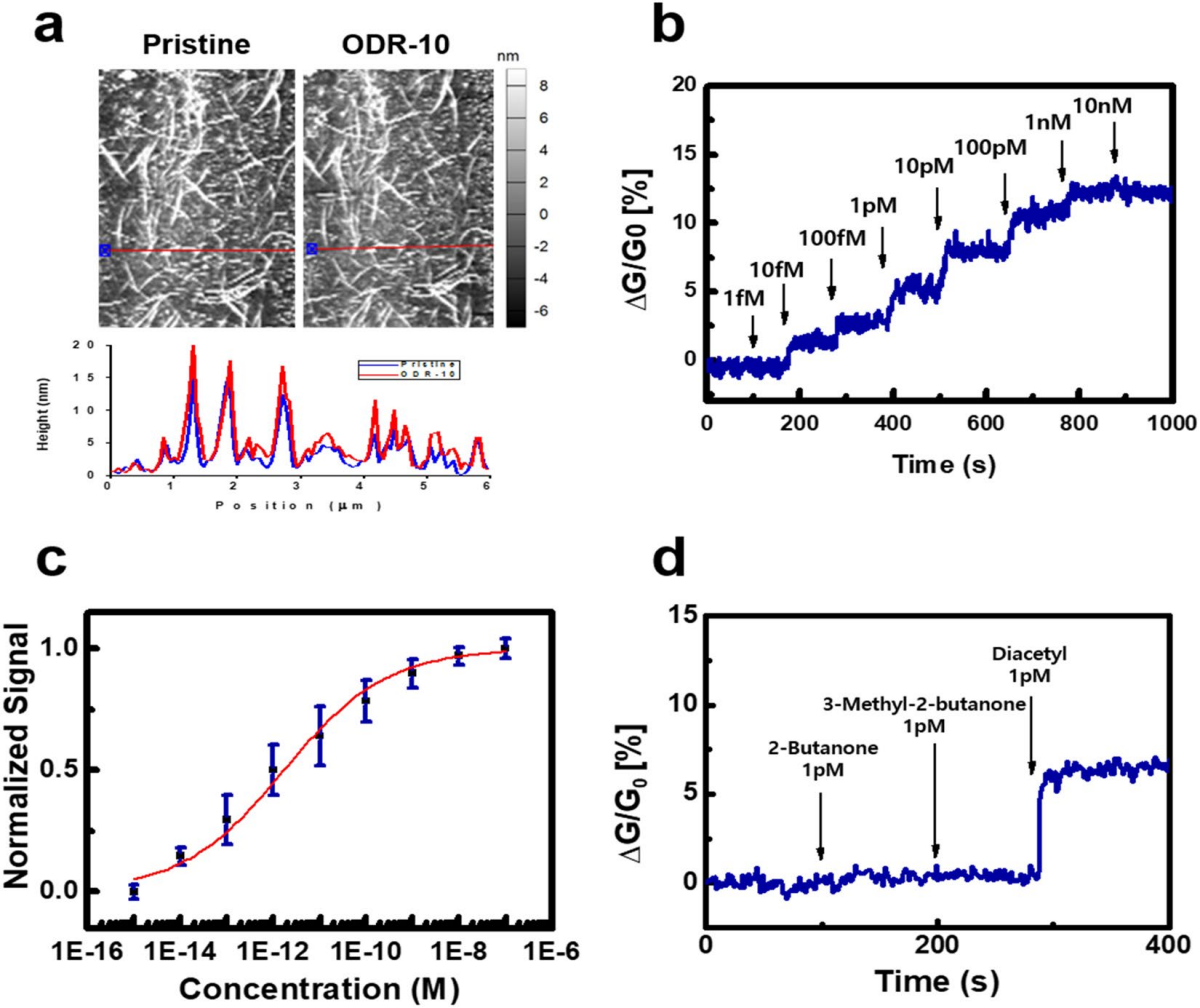
Responses of CNT-based bioelectronic noses to diacetyl solutions. (**a**) AFM images (top) of CNT channels areas before (marked by “Pristine”) and after (marked by “ODR-10”) immobilization of ODR-10 receptors and height profile comparison (bottom) of the areas before (blue) and after (red) immobilization. (**b**) Real-time electrical response of a CNT-based bioelectronic sensor to diacetyl solutions at various concentrations. (**c**) Normalized response of CNT-based bioelectronic sensors to diacetyl at various concentrations. (**d**) Real-time electrical response of a CNT-based bioelectronic sensor to various structurally-similar substances.

Figure 3a shows the atomic force microscopy (AFM) images of CNT channels before and after the immobilization of ODR-10 receptors. A silicon-based AFM tip was used in a non-contact mode with a typical scan speed of 0.5 μm/s. For comparison, the same location of the CNT channel *before* and *after* the receptor immobilization was imaged repeatedly. The average height along the 6 μm region increased by 2.9 ± 1.3 nm after the immobilization process. These results are similar to those reported previously, supporting the successful immobilization of receptors on the CNT channel^29,30^.

Figure 3b shows the real-time response of a CNT-based bioelectronic nose device to various concentrations of diacetyl in aqueous environments. The detailed experimental procedures are described in the Methods section. In brief, for the measurement, 9 μL of phosphate buffered saline (PBS) was placed on the CNT channel of a bioelectronic nose device. Then, the source-drain currents in the CNT channel were monitored in real-time during the introduction of diacetyl solutions with different concentrations ranging from 1 fM to 10 nM. Here, the relative change of conductance |*ΔG/G*_0_| in the CNT channels at a certain concentration was utilized as a sensor signal. The sensor signal began to increase at 10 fM diacetyl, indicating the high sensitivity of our sensor with a lower detection limit (LOD) than those of other previous methods^10,12,15,31^. The comparison of our method with previous methods is presented in a table (Fig. S2 in Supporting Information). Note that the bare CNT-FET without ODR-10 did not exhibit a conductance change by the addition of diacetyl (Fig. S3 in Supporting Information). These results indicate that the responses of the sensor signal could come from the selective binding between diacetyl and ODR-10 on the CNT channels. Such responses can be ascribed to the change of electrical charges of ODR-10 caused by the specific binding of diacetyl. Previously, GPCRs such as ODR-10 have been reported to be activated by the specific binding events between ligands and GPCRs, initiating the structural rearrangement of GPCRs, which result in the change of charge states in it^16,32^. Subsequently, the changed charge states in GPCRs gave the gate effects on the CNT channels, resulting in the conductance increase in CNT channels^32^.

Figure 3c shows the normalized responses of CNT-based bioelectric nose sensors to diacetyl with various concentrations. Here, the normalized responses were calculated by normalizing signals of sensors with respect to their maximal signal values at high concentrations. The measurements at a single concentration were repeatedly carried out by using four bioelectronic nose sensors to obtain average values and standard errors. Our sensors began to increase conductance from the concentration of 10 fM, and the responses were almost saturated at 100 nM diacetyl. Here, the error bars were smaller than the sensor responses at 10 fM diacetyl, indicating the reliable detection of diacetyl with high sensitivity.

The dose-dependent responses of bioelectronic nose sensors were analyzed by the Hill equation as reported previously^24,25,32^. Previous works showed that the normalized responses *N* of bioelectronic nose sensors based on a CNT-FET could be written like^24,25,32^

1 $$N=\frac{{C}^{n}}{\frac{1}{K}+{C}^{n}}$$

where C, K and n are *the concentration of diacetyl solution*, *the equilibrium constant for the binding of the diacetyl to ODR-10 receptors* and *a Hill’s coefficient*, respectively. The fitting of normalized responses by Eq. (1) allowed us to calculate the equilibrium constant K between diacetyl and ODR-10 as ~1.7 × 10^−12^ M in the diacetyl solution with PBS. The calculated K value is rather small compared with reported values using cell-based fluorescence assays (Fig. S2 in Supporting Information)^10,31^. Presumably, in case of cell-based assays, the binding event of odorant molecules onto olfactory receptors should go through several intermediate chemical processes to be detected *via* fluorescence signals, while, in our experiment, the activity of receptor protein was directly measured by a sensitive CNT-based transducer. We also observed similar results in previous works using other receptor proteins^16,17,26^. The result shows that our bioelectronic sensor system could respond to diacetyl at much lower concentrations than those of cell-based sensing systems. Similar trends were also previously reported in case of other bioelectronic nose sensors^24,25^. The plausible explanation may be that our device could directly detect the binding of receptor proteins, ODR-10, without any intermediate biological steps, resulting in high sensitivity of our method. However, the cell-based fluorescence assays relied on complicated signal transduction steps inside the cell^33–35^. In this case, such intermediate steps in cell-based systems require several different materials other than diacetyl to generate sensing signals, resulting in low sensitivity of cell-based systems. The Hill coefficient n was calculated as 0.32 for the binding of diacetyl to ODR-10 in detergent micelles, which is similar to the values reported previously^36^. Note that the low Hill coefficient (n < 1) implies the negatively-cooperative binding of diacetyl to ODR-10. It was previously reported that GPCRs can form dimers, or higher order oligomers. In such a case, the binding of ligand molecules can deform the proteins, leading to the negatively-cooperative binding^37,38^. Presumably, in our works, the binding of diacetyl to one ODR-10 receptor site could induce the deformation of the receptor, resulting in remaining sites to bind diacetyl with a lower affinity.

Figure 3d shows the real-time response of a CNT-based bioelectronic sensor to various substances. Here, we consecutively added the 1 pM solutions of 2-butanone, 3-methyl-2-butanone and diacetyl (2,3-butanedione) to the CNT-based bioelectronic sensor while measuring the sensor responses. The 2-butanone and 3-methyl-2-butanone molecules have similar structures to diacetyl molecules, while they do not have a butter flavor (Fig. S4, Supporting Information)^8^. Note that the addition of 2-butanone and 3-methyl-2-butanone solutions with 1 pM concentrations caused the negligible conductance changes, while the addition of a diacetyl solution with a 1 pM concentration sharply increased the conductance of the CNT channel in the sensor. The result clearly shows that our CNT-based bioelectronic nose could discriminate diacetyl from other similar molecular species with a high selectivity.

### Detection of diacetyl in various commercial alcoholic beverages using CNT-based bioelectronic noses

**Figure 4:**
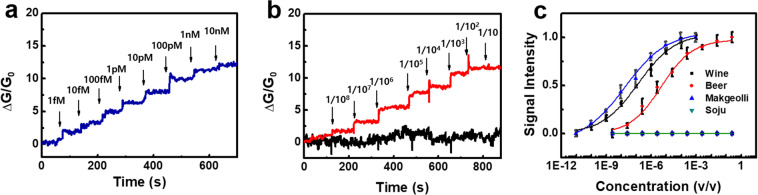
Detection of diacetyl in various commercial alcoholic beverages using CNT-based bioelectronic noses. (**a**) Real-time responses of a CNT-based bioelectronic sensor to various concentrations of diacetyl added to “soju”, a Korean style liquor. The addition of soju-diacetyl solutions to the sensor caused immediate increases in the conductance of the bioelectronic nose with ODR-10 in a dose-dependent manner. (**b**) Real-time responses of CNT-based bioelectronic noses to diacetyl in beer and soju solutions. When diluted beer solutions were introduced, the conductance of a CNT channel increased in a dose-dependent manner. However, the sensor did not exhibit conductance changes when diluted soju was introduced. (**c**) Normalized responses of CNT-based bioelectronic sensors to various commercial alcoholic beverages. The x-axis (v/v) represents the volume/volume percent of the alcoholic beverages in the PBS buffer solution.

To demonstrate the feasibility of a CNT-based bioelectronic sensor for practical applications, we also conducted the experiments of detecting diacetyl in commercial alcoholic beverage samples. Figure 4a shows the real-time response of a CNT-based bioelectronic nose to diacetyl added to Korean style liquor called “soju”. Soju, traditionally made from rice, is a distilled alcoholic beverage which is known to contain almost no diacetyl^39^. We added different amounts of diacetyl to soju and demonstrated that our sensors can detect diacetyl in alcohol-based beverage environments (see the details in the Methods section). For the sensing experiments, drain-source currents of the sensors were measured using a semiconductor analyzer while adding various concentrations of soju-diacetyl mixture solutions. The drain-source bias voltage was maintained as 0.1 V during the measurement. The addition of soju-diacetyl solutions to the sensor caused immediate increases in the conductance of the CNT-FET with ODR-10 in a dose-dependent manner, which is in a good agreement with the results in Fig. 3b. The results show that our sensor could detect diacetyl in real-food environments such as alcoholic beverages.

Figure 4b shows the real-time responses of a CNT-based bioelectronic nose to diacetyl in real-alcoholic beverages of commercial beer and soju. Beer and soju samples were prepared by means of the dilution of commercial beer and soju with PBS buffer solutions (see the details in the Methods). The addition of the beer samples caused immediate increases in the conductance of the bioelectronic nose in a dose-dependent manner, while the sensor did not exhibit a conductance change after the addition of soju samples. This is because soju is a distilled alcoholic beverage which contains almost no diacetyl^39^. We also confirmed that a *bare* CNT-based sensor without ODR-10 did not respond to beer samples (Fig. S5 in Supporting Information). These results clearly indicate that the CNT-based bioelectronic sensors can detect diacetyl in complex real-beverage environments such as beer with a high sensitivity.

**Figure 5:**
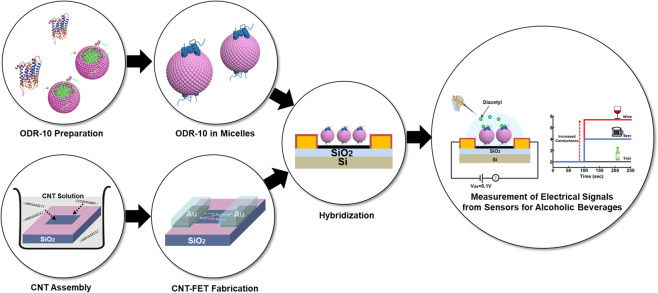
Graphical abstract.

Figure 4c shows the normalized responses of CNT-based bioelectronic sensors to various alcoholic beverage samples at different concentrations. Detailed methods for preparing wine, beer, “makgeolli” and soju samples are described in the Methods section. Makgeolli is a fermented Korean wine which is known to contain diacetyl^40^. Each data point was obtained by multiple measurements using four or more bioelectronic nose devices (Fig. S6 in Supporting Information). In the case of soju samples, sensors did not show responses. However, the sensors began to respond to the addition of wine and makgeolli samples with the diluted concentration of 10^−10^, and their signals were saturated at the diluted concentration of 10^−3^. For beer samples, the sensor began to show responses from the diluted concentration of 10^−8^, and sensor signals were saturated at the diluted concentration of 10^−1^. We also confirmed that the *bare* CNT-FET without ODR-10 exhibited negligible conductance changes after the addition of diluted wine and makgeolli samples (Fig. S7 in Supporting Information). The results clearly show that the responses of our bioelectronic nose came from the specific binding between diacetyl and ODR-10. Since our method directly detected diacetyl which bound to ODR-10, it can be a convenient method to efficiently evaluate the content of diacetyl in real-alcoholic beverages. By fitting the dose-dependent response data using Eq. (1) and the estimated equilibrium constant *K*_*diacetyl*_ *of 1.7 × 10*^*−12*^ M for the pure diacetyl solution (Fig. 3c), we could estimate the levels of diacetyl in the commercial *beer*, *wine* and *makgeolli* as *4.2 × 10*^*−7*^ M, *1.2 × 10*^*−5*^ M, and *2.6 × 10*^*−5*^ M, respectively. Note that these measured diacetyl concentrations are close to the previously reported values^1,2,40^. The result clearly shows that our sensor could quantitatively evaluate diacetyl in complex environments such as real-beverage products. Presumably, micelle structures stabilized ODR-10 receptors, enabling the stable operation of our bioelectronic nose devices in complex environments such as alcoholic beverages. Since the long-term stability of a biosensor is usually determined by the stability of receptor proteins used in the sensor, we can also expect that the stabilization of ODR-10 receptors in micelle structures should enhance the lifetime of our bioelectronic nose devices. Previous works in our group show that bioelectronic nose devices based on GPCR proteins stabilized in similar lipid nanostructures could be used under gaseous ambient conditions for more than 6 weeks^41^. The entire work flow chart of this study is shown in Fig. 5.

## Conclusion

We have developed a highly sensitive and selective bioelectronic nose using ODR-10 receptors stabilized in micelles for the detection of diacetyl in real-alcoholic beverages. In this work, the overexpressed ODR-10 was purified and reconstituted in detergent micelles to build a stable structure in an aqueous environment, and they were immobilized on a CNT-FET to fabricate a bioelectronic nose device. Our sensor could detect diacetyl with a detection limit (LOD) of 10 fM, which is more sensitive than previous methods such as fluorescence assays (LOD ~0.1 μM) and HPLC (LOD ~10 nM) (Fig. S2 in Supporting Information)^12,31^. In addition, our sensors also discriminate diacetyl from other structurally similar substances in different environments. Interestingly, we could demonstrate the detection of diacetyl directly in various fermented alcoholic beverages such as beer, wine and makgeolli. In this regard, our sensor can be a useful tool for versatile industrial applications such as the evaluation of alcoholic beverages and some fermented foods.

## Methods

### Materials

Single-walled semiconducting 99% carbon nanotubes (CNTs) were purchased from NanoIntegris Inc. (USA) and used as the channel materials of our sensors. Diacetyl, 2-butanone, 3-methyl-2-butanone and other chemical reagents were purchased from Sigma Aldrich (USA) and used as received. Various commercial alcoholic beverages of beer, wine, makgeolli and soju were purchased from a grocery store in Korea.

### Cloning, expression, and purification of ODR-10

Cloning, expression, and purification of ODR-10 were performed using similar methods those in a previous study^26^. *ODR-10* was amplified using polymerase chain reaction (PCR), and the amplified product was inserted into the pET-DEST42 vector (Invitrogen) using the pENTR/D-TOPO cloning kit (Invitrogen) and recombination reaction of LR Clonase (Invitrogen). The pET-DEST42 vector harboring *ODR-10* was used for transformation of *E. coli* strain BL21 (DE3). After the seed inoculum of *E. coli* was cultured in Luria–Bertani medium with 50 μg/mL ampicillin at 37 °C overnight, 1 mM isopropyl β-d-thiogalactoside was added to induce *ODR-10* expression in the main culture. The cells were cultured for 4 h and harvested by centrifugation (7,000 × *g*, 20 min, 4 °C), after which they were suspended and sonicated for 10 min to cause cellular disruption. Proteins were collected by centrifugation (7,000 × *g*, 20 min, 4 °C) and solubilized in a buffer containing 0.1 M Tris-HCl (pH 8.0), 1 mM EDTA, 20 mM sodium dodecyl sulfate (SDS), and 0.1 M dithiothreitol. The solubilized proteins were dialyzed against 100 mM sodium phosphate (pH 8.0) and 10 mM SDS. The ODR-10 was purified using a Ni^2+^ column (GE Healthcare). The concentration of ODR-10 was measured using a bicinchoninic acid (BCA) assay kit (Thermo Scientific). ODR-10 expression was analyzed by SDS-PAGE and western blotting using the anti-V5-probe antibody (Thermo Scientific).

### Reconstitution of purified ODR-10

The reconstitution process of purified ODR-10 was similar to that in a previous study^26^. The purified protein sample was dialyzed in a buffer containing 0.1 M Tris-HCl (pH 8.0), 0.5 mM EDTA, and 10 mM SDS, which was diluted to 0.1 M Tris-HCl (pH 8.0), 0.5 mM EDTA, and 3 mM SDS through dialysis. Then, 6 mM n-dodecyl-β-d-maltopyranoside (DDM), 1 mM glutathione disulfide 6 mM 6-cyclohexylhexyl-β-d-maltoside (Cymal 6), 5 mM glutathione, and 6 mM methyl-β-cyclodextrin were added to the dialyzed protein and frozen at −20 °C for 48 h. CaCl_2_ (25 mM) was added after the frozen sample was thawed at 4 °C. After overnight incubation at 4 °C, the sample was dialyzed against a buffer containing 0.1 M Tris-HCl (pH 7.4), 1 mM EDTA, 300 mM NaCl, 1 mM Cymal 6, and 1 mM DDM. The reconstituted sample was stored at 4 °C.

### Circular dichroism (CD) spectrum measurements

The process of CD spectrum measurements followed previously reported methods^26^. The reconstituted sample was diluted with a buffer containing 0.1 M Tris-HCl (pH 7.4), 1 mM EDTA, 300 mM NaCl, 1 mM Cymal 6, and 1 mM DDM. The CD spectra of the reconstituted sample and buffer were scanned between 190 and 260 nm using a Chirascan-Plus CD spectrometer (Applied Photophysics). After the background signal was subtracted, the secondary structure of reconstituted ODR-10 was analyzed.

### Cell culture

Cell culture was performed in a similar way to that in a previous study^26^. Human embryonic kidney-293 (HEK-293) cells were cultured in Dulbecco’s modified Eagle medium (DMEM) containing 10% fetal bovine serum and 1% penicillin−streptomycin (Gibco) at 37 °C in an atmosphere of 5% CO_2_. The cells were transfected with pcDNA3 containing ODR-10 using Lipofectamine 3000 (Invitrogen) following the manufacturer’s instructions.

### Intracellular calcium assay

The procedure of intracellular calcium assay was similar to that in a previous study^26^. The calcium indicator dye, Fluo-4 AM (Molecular Probes) was incorporated into HEK-293 cells expressing ODR-10 *via* addition of Fluo-4 AM and 1 mM probenecid (Molecular Probes) in the cell culture medium following the manufacturer’s instructions. After the cells were incubated for 30 min at 37 °C and in a 5% CO_2_ atmosphere, the calcium indicator was washed with cell culture medium containing 1 mM probenecid. The cells were incubated for 30 min at 37 °C in a 5% CO_2_ atmosphere to reduce leakage of Fluo-4 caused by the intracellular esterase-mediated hydrolysis of Fluo-4 AM. 2,3-butanedione was added to the cell culture medium and fluorescence signal was measured using a spectrofluorophotometer (Tecan) with an excitation wavelength of 488 nm and an emission wavelength of 535 nm.

### Fabrication of CNT-based bioelectronic sensors

CNT-based bioelectronic sensors were fabricated as reported previously^32^. In brief, single-walled semiconducting CNTs were dispersed in 1,2-dichlorobenzene using an ultrasonic vibration for 4 h to the final concentration of 0.05 mg/mL. The patterns of octadecyltrichlorosilane (OTS) self-assembled monolayer (SAM) were formed on a SiO_2_ substrate (300 nm) using a conventional photolithography method as reported previously^32^. The patterned substrate was immersed into the CNT solution for 10 s and rinsed with 1,2-dichlorobenzene. Here, CNTs were selectively adsorbed onto the bare SiO_2_ regions without OTS SAM, and they formed the film of randomly-oriented CNT networks. Metal electrodes (Pd/Au 10 nm/15 nm) were fabricated using photolithography processes including thermal evaporation and a lift-off method. The source and drain electrodes were then passivated with a photoresist to prevent leakage currents during the electrical measurements in liquid environments. The exposed CNT channel area had a width of 3 μm and a length of 10 μm. Our fabrication method enables us to mass-produce devices in a wafer scale.

### ODR-10 receptors immobilization onto CNT-based bioelectronic sensors

A 1-Pyrenebutanoic acid N-hydroxysuccinimidyl ester (PSE, Molecular Probe) solution (1 mM in methanol) was applied onto the fabricated CNT-based bioelectronic sensors for 1 h at a room temperature and washed with fresh methanol. In this strategy, the pyrene moieties of PSE molecules interacted with the sidewalls of CNTs *via* π-stacking^17^. Then, the CNT-based sensors were incubated in the solution including ODR-10 receptors for 4 h at 4 °C so that the ODR-10 receptors were selectively attached onto the PSE layer on the CNT channel region of the CNT-based sensors. In addition, succinimidyl groups of the PSE molecules bound to proteins intercalated in the receptors.

### Substances preparations

Diacetyl, 2-butanone and 3-methyl-2-butanone were dissolved in PBS buffer solutions. The beer, wine and makgeolli were diluted at different ratios with PBS buffer solutions.

### Electrical measurements

For measuring the responses of a CNT-based bioelectronic nose to various substances, CNT-based sensors functionalized with ODR-10 in detergent micelles were connected to a Keithley 4200 semiconductor analyzer. Source-drain bias voltage of 0.1 V was applied and maintained during electrical measurements. A 9 μL droplet of the PBS buffer was placed on the CNT channel of the sensor, and source-drain currents were monitored upon the addition of various concentrations of diacetyl solutions and reagents.

## Supplementary information

Supplementary Information.
