# Supplementary material for: Micelle-stabilized Olfactory Receptors for a Bioelectronic Nose Detecting Butter Flavors in Real Fermented Alcoholic Beverages
Source: Sci Rep. 2020 Jun 3;10:9064. doi: 10.1038/s41598-020-65900-6 (PMC7270175; doi:10.1038/s41598-020-65900-6)
Supplement: Supplementary file 1 — Supplementary Information. [file 41598_2020_65900_MOESM1_ESM.pdf]

SUPPLEMENTARY MATERIAL FOR:

Micelle-stabilized Olfactory Receptors for a Bioelectronic Nose Detecting Butter Flavors in Real Fermented Alcoholic Beverages

Narae Shin[1,+] Seung Hwan Lee[2,3,+], Viet Anh Pham Ba[1,4], Tai Hyun Park[2,*], and Seunghun Hong[1,*]

[1]Department of Physics and Astronomy, and Institute of Applied Physics, Seoul National University, Seoul 08826, Korea
[2]School of Chemical and Biological Engineering, Institute of Chemical Processes, Seoul National University, Seoul 08826, Korea
[3]Department of Bionano Engineering and Bionanotechnology, Hanyang University, Ansan 15588, Korea
[4]Department of Environmental Toxicology and Monitoring, Hanoi University of Natural Resources and Environment, Hanoi, Vietnam

* seunghun@snu.ac.kr, thpark@snu.ac.kr

SUPPORTING FIGURE

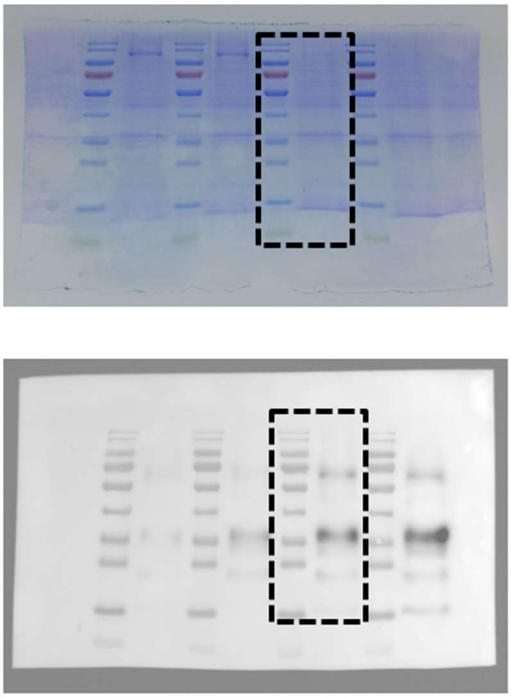

Full-length gel
corresponding Figure 2a (left)

Full-length blot
corresponding Figure 2a (right)

Figure S1. Full-length gel and blot analysis of purified ODR-10 produced in *E. coli*.

Analytical method	Detection limit	Hill Coefficient	Reference
**Cell based SPR**	**10 µM**	**-**	**10**
**HPLC**	**10 nM**	**-**	**12**
**Voltammetry**	**10 nM**	**-**	**15**
**Cell-based Fluorescence Method**	**100 nM**	**2.25 µM**	**29**
**CNT-FET**	**10 fM**	**1.7 pM**	**This work**

Figure S2. Table. Detection limit values for various analytical methods.

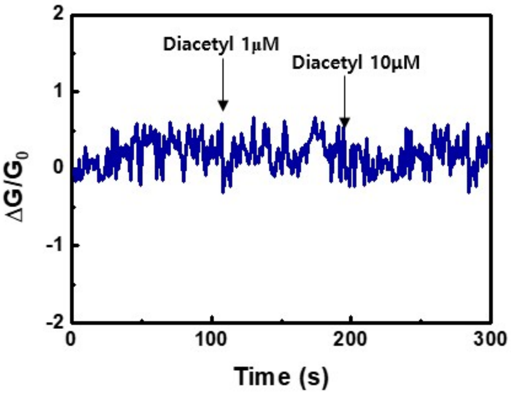

Figure S3. Real-time responses of a bare CNT-based sensor without ODR-10 receptors to diacetyl in PBS solutions. Diacetyl did not cause the conductance change.

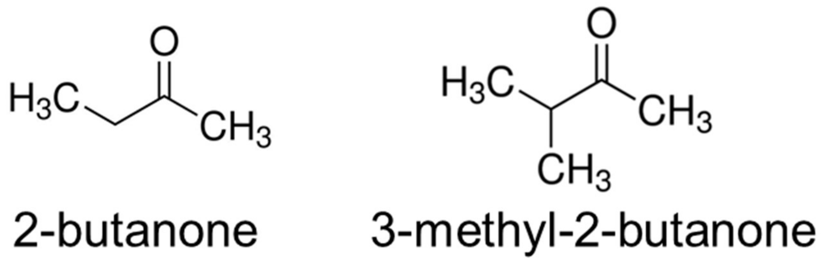

Figure S4. Structures of two different odorants, 2-butanone and 3-methyl-2-butanone.

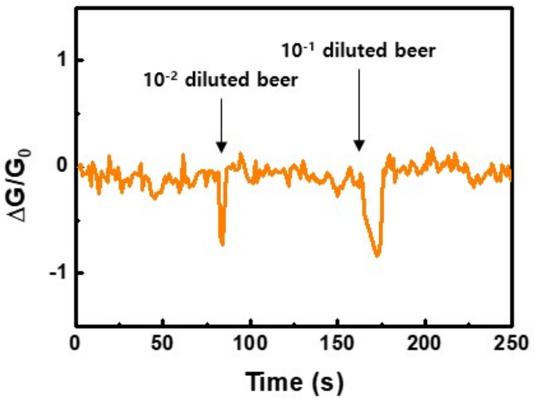

Figure S5. Real-time responses of a bare CNT-based sensor without ODR-10 receptors to diluted beer in PBS solutions. Diluted beer solutions did not cause the conductance change of the bare CNT channel in the sensor.

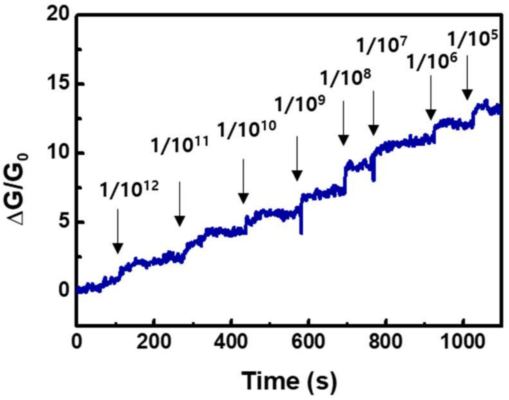

Figure S6. A real-time response of CNT-based bioelectronic sensors with ODR-10 to diacetyl in diluted wine solutions. When diluted wine solutions were introduced, the conductance of a CNT channel increased in a dose-dependent manner.

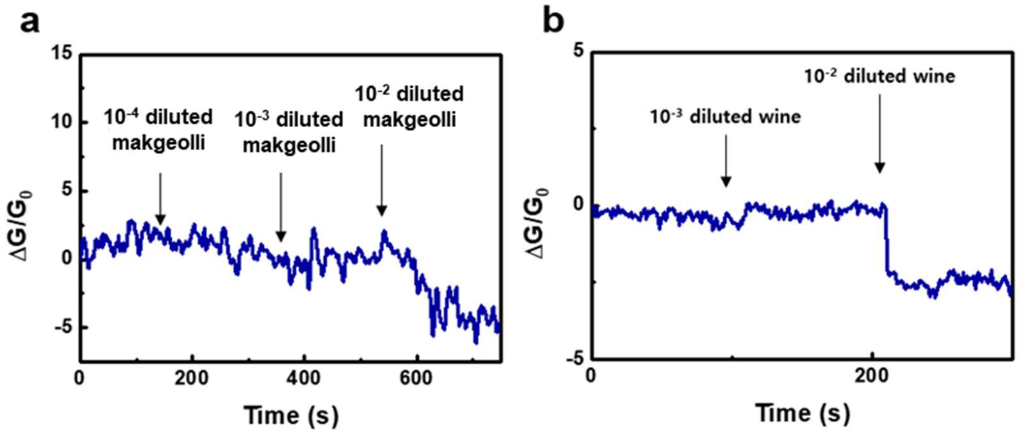

Figure S7. Real-time responses of CNT-based bioelectronic sensors without ODR-10 to diacetyl in diluted makgeolli and wine solutions. The addition of diluted wine and makgeolli solutions resulted in the conductance change at diluted concentration of 10^{-2}.
